# Subjective norms of paintings: Integrating perceptual, cognitive, and emotional dimensions

**DOI:** 10.3758/s13428-026-03107-9

**Published:** 2026-07-07

**Authors:** Khaoula Ennahli, Cristiane Souza, Margarida Vaz Garrido

**Affiliations:** Iscte-Instituto Universitário de Lisboa, Avenida das Forças Armadas, 1649-026 Lisboa, Portugal

**Keywords:** Paintings, Norms, Perceptual dimensions, Cognitive dimensions, Emotional dimensions

## Abstract

Artworks, such as paintings, are frequently utilized as stimuli in research and interventions. However, the complexity of their perceptual, emotional, and cognitive properties necessitates rigorous validation to ensure the reliability of research outcomes and the efficacy of their application in real-world contexts. A few studies have already established norms for paintings. Still, the type of stimuli (e.g., artificial intelligence [AI]-generated) and the comprehensiveness of the assessed dimensions may not entirely capture the complexity of human-made ecological artworks. The current study establishes norms for a diverse set of 144 human-made paintings retrieved from the publicly accessible WikiArt database and thematically clustered into four categories: objects (*n* = 36), places (*n* = 36), people (*n* = 36), and abstract (*n* = 36) works. European Portuguese native speakers (*N* = 361) rated the paintings in 11 perceptual, cognitive, and emotional dimensions. Participants also provided qualitative descriptions of the paintings, detailing their content and recognized elements. Descriptive data for each painting are provided by dimension and category. Correlations among the dimensions and between individual variables (e.g., personality traits, prior experience with the arts) and the evaluative ratings are also reported. The results indicate that this painting set is diverse, allowing for the selection of artworks that represent different levels of the evaluated dimensions across stimulus categories. These norms fill a critical gap in the standardized evaluation of paintings, facilitating a more precise and controlled use of these stimuli in experimental, clinical, and creative expression applications.

## Introduction

Art, in its myriad forms, has long been recognized for its profound impact on human experience. The defining attribute of art is its communicative and mobilizing power, as suggested by the well-known aphorism “a picture is worth a thousand words.” It is widely acknowledged that art possesses the power to unsettle, provoke, or deeply move individuals, thereby evoking what is often regarded as an aesthetic experience. Yet, defining the nature and characteristics of aesthetic experience remains a remarkably contentious exercise (Chatterjee & Vartanian, 2014).

Aesthetic experience involves a complex interaction of perceptual, cognitive, and emotional systems and processes (Chatterjee, 2003; Leder et al., 2004). Specifically, viewing paintings has been shown to engage a distributed network within the brain, involving regions associated with visual processing (e.g., the occipital lobes), object and scene recognition (e.g., ventral temporal structures), emotional response (e.g., the anterior insula), and internal mental processes like the integration of information and inner-oriented cognition (e.g., the posterior cingulate cortex; Vartanian & Skov, 2014).

Behavioral research consistently demonstrates that aesthetic experiences offer cognitive benefits for healthy individuals. For example, extended exposure to art enhances recognition memory accuracy (Daviddi et al., 2022). Also, compared with real pictures, artworks can evoke memories, often leading to more detailed recollection or to vivid personal memories triggered by the visual stimulus (Mastandrea et al., 2019; Tinio & Leder, 2009). Additionally, paintings (especially abstract or thought-provoking works) can elicit emotional arousal (Pelowski et al., 2017), which may enhance memory formation and retrieval, as well as other cognitive processes (e.g., Phelps & Sharot, 2008). Art exposure has also been found to boost creativity: participants perform better on divergent-thinking tasks after viewing paintings (Forgeard & Elstein, 2014; Leder et al., 2004). Empirical studies have also documented that art engagement, whether participatory or observational, enhances mood, health, and overall well-being across various age groups (Averbach & Monin, 2022; Mastandrea et al., 2019; Roswiyani et al., 2019).

Art-based interventions are also effective in alleviating anxiety and depression in clinical settings (e.g., oncology settings), promoting coping skills, self-expression, and social interaction (Geue et al., 2010). Cognitive enhancements have also been noted. For example, evidence suggests that structured art-viewing in museums boosts memory processes. Specific benefits include enhanced episodic memory among individuals with dementia (Eekelaar et al., 2012). Additionally, integrating creative art therapy with standard physical therapy in stroke patients led to significantly greater improvements in global cognitive function (Kongkasuwan et al., 2016). Likewise, in a pilot randomized controlled trial, Yun et al. (2023) found that arts and crafts therapy notably improved executive functions (Yun et al., 2023). Such cognitive enhancement likely stems from the engagement of higher mental processes, including attention, cognitive control, and memory, during aesthetic experiences (Mullennix, 2019).

Research into cognitive and emotional reactions to paintings has revealed intricate interactions between the characteristics of the artworks and the viewers' experiences. Emotional responses to visual art are shaped by its content and style. Artworks with negative themes may evoke mixed emotions, particularly sadness accompanied by feelings of being moved, and occasionally even joy, rather than distress alone (Nummenmaa & Hari, 2023). Additionally, visual complexity and novelty increase interest and arousal, especially when the viewer can make sense of the artwork (Husselman et al., 2024; Marin et al., 2016). Familiarity also enhances pleasure, though novelty may be preferred in abstract or complex works (Song et al., 2021).

The style of the paintings is also critical in shaping preferences among untrained viewers, with realistic styles often outweighing aspects such as color and subject matter (Hardiman & Zernich, 1982). Prior work by Marković (2011) showed that representational paintings tend to receive higher ratings on specific perceptual dimensions, such as form and complexity, as well as on semantic dimensions related to the depiction of a recognizable external reality, and on affective dimensions. Conversely, abstract art, particularly abstract paintings, elicits more individual contributions than representational paintings, as reflected in variability in both brain responses and semantic meaning generation (Durkin et al., 2022).

Importantly, individual variables such as personality, experience with visual arts, age, and gender can shape cognitive and emotional reactions to art. For instance, individuals with high openness to experience tend to exhibit greater cognitive and emotional engagement with abstract art (Chamorro-Premuzic et al., 2010). In contrast, characteristics such as conscientiousness and agreeableness are associated with a preference for structured, representational artworks, including portraits and object-focused paintings (Furnham & Walker, 2001). Furthermore, neuroticism appears to be associated with a preference for art that elicits strong emotional responses, often evident in human-centric paintings (Rawlings, 2003). Prior experience with the arts may also influence how viewers attend to, interpret, and evaluate artworks, as art training and expertise have been associated with differences in visual-spatial processing and top-down attentional strategies (Chamberlain et al., 2019; Morrone & Pedlar, 2024). Studies have also identified age-related differences in art preferences, with younger adults generally favoring contemporary or abstract art, while older adults tend to prefer traditional or figurative art (Leder et al., 2004; Silvia, 2006). Gender differences have also been documented, with women typically exhibiting stronger emotional reactions and greater empathy towards portraits or art that depicts interpersonal themes (McManus & Furnham, 2006).

Despite cumulative evidence from various research fields, a notable gap remains in the standardization of stimuli used in art-related research. To date, most studies still rely on non-normalized stimuli (e.g., Eekelaar et al., 2012; Vessel et al., 2012, 2019). As a result, observed effects may partly reflect uncontrolled stimulus variation rather than the intended manipulation, thereby hampering the comparability and replicability of research findings across studies and cultures (Chatterjee & Vartanian, 2016; Pelowski et al., 2017). Developing a normative database of paintings addresses this problem by allowing researchers to preselect matched subsets of stimuli and distribute them across conditions while minimizing confounding variation. In this way, standardized norms can strengthen internal validity, improve reproducibility, and facilitate cumulative comparisons across studies.

Although standardized norms have been developed for various types of visual stimuli, such as objects (e.g., Brodeur et al., 2010; Souza et al., 2021), symbols (e.g., Snodgrass & Vanderwart, 1980; Prada et al., 2016), emojis (e.g., Rodrigues et al., 2018), and emotional expressions (Garrido & Prada, 2017; Goeleven et al., 2008), and affectively rated images (e.g., Lang et al., 2008), less attention has been given to art-related stimuli. The idea that the subjective nature of art renders it incompatible with scientific inquiry has likely contributed to this continued neglect. However, developing and applying standardized norms for artworks could help bridge the gap between subjective aesthetic experience and objective empirical analysis, thereby enabling more consistent and replicable research outcomes.

To our knowledge, only two standardized databases are currently available for experimental research with visual artworks. The Vienna Art Picture System (VAPS; Fekete et al., 2022) offers an extensive collection of 999 fine art paintings, encompassing five genre categories (portraits, scenes, landscapes, still-lifes, and abstracts) and 13 historical styles, ranging from the Renaissance to contemporary art. Each painting was normed on five affective and perceptual dimensions—valence, arousal, liking, familiarity, and visual complexity—offering a validated foundation for the systematic selection of stimuli in empirical aesthetics and cognitive research. Although the VAPS represents the first large-scale standardized set of genuine artworks, its scope primarily targets affective variables, complemented by a single perceptual measure (visual complexity), leaving aside cognitive, associative, and mnemonic dimensions that are also central to neurocognitive assessment and intervention. In parallel, the art.pics database (Thieleking et al., 2020) provides artificial intelligence (AI)-generated, art-like images of common objects. While this approach ensures experimental control through simplified features and balanced sets, such computer-generated stimuli may not capture the expressive, contextual, and cultural richness of human-made art. The oversimplified and synthetic visual features of art.pics might constrain the range of perceptual and emotional responses they elicit. Although both databases provide valuable normative tools, they overlook important dimensions and potential art-related experiences.

The current study seeks to address these gaps by developing a comprehensive set of standards encompassing 11 perceptual, cognitive, and emotional dimensions involved in evaluating pictures of real human-made paintings. Additionally, these norms further expand existing databases by including four categories—people, objects, places, and abstract paintings—a theoretically motivated distinction grounded in research showing that visual experience is structured by meaningful semantic categories, with people, objects, and places engaging partly dissociable perceptual and memory-related processes (e.g., Anderson et al., 2021; Jorge et al., 2018; Peelen et al., 2023; Souza et al., 2026). Abstract paintings were included to extend the database beyond representational content and to provide a comparison category characterized by lower semantic specificity. Moreover, from the 11 dimensions assessed, two included open-ended questions, allowing for both quantitative ratings and qualitative insights. These dimensions provide a deeper understanding of the complexities of psychological and aesthetic experiences. The resulting standards aim to lay the groundwork for subsequent studies, thereby enhancing the consistency and reliability of research that uses images of paintings as stimuli.

### Dimensions of interest

Paintings, as complex visual compositions, engage multiple layers of perception, affect, and cognition. They often incorporate recognizable or abstract forms, vivid colors, and intricate patterns, evoking emotional responses and personal associations in viewers. Thus, the dimensions and rating scales employed here, drawn from general principles in perception, cognition, and emotion research, provide a framework for systematically examining how people perceive, understand, and respond to paintings.

#### Visual complexity

Visual complexity refers to the degree of detail or intricacy within a painting, including aspects such as color, shape, and contrast (Snodgrass & Vanderwart, 1980). Visual complexity often correlates negatively with familiarity, as less familiar items appear more complex (Brodeur et al., 2012; Prada et al., 2016; Souza et al., 2021). Complexity influences visual search and categorization processes, with more intricate stimuli generally requiring longer processing times (Alario & Ferrand, 1999; McDougall et al., 1999). Higher visual complexity also correlates with arousal and aesthetic appeal (Souza et al., 2021). This dimension measures the level of detail, intricacy, and visual information, such as patterns, textures, and color diversity, displayed in the painting.

#### Concreteness

This dimension assesses the degree to which a painting represents real-world objects, materials, or people rather than abstract, non-representational, or highly abstract forms (McDougall et al., 1999; Prada et al., 2016). Concreteness correlates positively with familiarity and meaningfulness (Prada et al., 2016). Concrete representations are more detailed and often easier to interpret, though this advantage may decrease with experience (e.g., McDougall et al., 1999; McDougall & Reppa, 2008). This dimension captures how concrete, understandable, and perceptually recognizable the painting’s content is.

#### Aesthetic appeal

This dimension reflects how visually pleasing individuals find a given painting (e.g., McDougall & Reppa, 2008; Prada et al., 2016; Souza et al., 2021). It is closely linked to familiarity and visual complexity, as familiar and visually simple stimuli are generally more appealing than unfamiliar, complex ones (McDougall & Reppa, 2008). In visual search tasks, aesthetic appeal enhances performance for complex stimuli but has no notable effect on performance for simple stimuli (Reppa et al., 2008). This dimension captures the degree to which the painting is perceived as visually pleasing or beautiful, informed by its composition, color, and form.

#### Valence

Valence captures the intrinsic attractiveness or aversiveness of a stimulus, reflecting its positive or negative nature (Frijda, 1986; Prada et al., 2016; Souza et al., 2021). It shapes perception, memory, and judgment, with stimuli strong in valence eliciting more intense emotional responses and improving recognition performance (Adelman & Estes, 2013). While it may overlap with aesthetic appeal, valence remains distinct and crucial for understanding emotional engagement. This dimension captures the emotional valence of the painting, ranging from negative to positive, and its impact on emotional responses.

#### Arousal

Arousal measures the degree of activation elicited by a stimulus, distinguishing states ranging from calm to intense (Prada et al., 2016; Souza et al., 2021). It often correlates with valence, typically following a U-shaped curve in which highly positive or negative stimuli elicit greater arousal (Lang et al., 1998). Arousal is essential for understanding emotional responses. This dimension reflects how energetically the painting engages the viewer, from a serene state to a highly stimulating experience.

#### Felt involvement

Involvement refers to the personal relevance an object or issue holds for an individual (Sherif & Sherif, 1967). Its importance has been demonstrated, for example, in consumer behavior research (Zaichkowsky, 1985), where felt involvement serves as a motivational state that influences attention, comprehension, and cognitive elaboration (Celsi & Olson, 1988). It is driven by intrinsic personal relevance and distinct from domain knowledge (Celsi et al., 1992). Felt involvement helps explain discrepancies between attitudes and behaviors in areas such as organic food consumption and leisure activities, although its temporal stability and sociodemographic correlations warrant further exploration (Havitz & Dimanche, 1999; Tarkiainen & Sundqvist, 2009). This dimension reflects the extent to which the painting evokes a personal connection and emotional engagement in the viewer, influencing their level of interest and attention*.*

#### Familiarity

Familiarity refers to the frequency with which an individual encounters or thinks about a given painting, influencing comprehension and usability (McDougall et al., 1999; Prada et al., 2016; Souza et al., 2021). Familiar images are generally more liked (Garrido & Prada, 2017; Souza et al., 2021) and tend to improve performance and mitigate other factors, such as complexity (Christ & Corso, 1982; Isherwood et al., 2007). Due to the difficulty in obtaining objective frequency data, subjective familiarity ratings are commonly used. Familiarity is influenced by factors such as age, native language, and social context (Pompéia et al., 2001). This dimension gauges whether the viewer recognizes or has prior knowledge of the painting.

#### Creative inspiration

Creative inspiration is a motivational state fueling the realization of creative ideas, often arising after initial idea generation (Thrash et al., 2010). It predicts creativity across multiple domains, mediating the relationship between idea generation and its ultimate expression. Aesthetic experiences often evoke inspiration, linking higher aesthetic appeal to enhanced creative output in tasks such as writing (Welke et al., 2023). Moreover, inspiration is “contagious,” influencing both creators and audiences, particularly those high in openness to experience (Ishiguro & Okada, 2020; Thrash et al., 2010). This dimension captures the painting’s ability to stimulate creative thinking, imagination, or inspiration in the viewer.

#### Memory evocation

Memory evocation examines the extent to which a painting triggers personal associations or recollections (Herz & Cupchik, 1995). Such memories can intensify emotional engagement, and art expertise can enhance recall and recognition of details (Vogt & Magnussen, 2007). This dimension measures how effectively a painting conjures personal memories, associations, or reminiscences.

#### Subjective interpretation

Interpretation involves attaching meaning to stimuli, influenced by personal perspectives and cultural backgrounds (Smythe, 1992). In art, subjective interpretation highlights the interplay between an artist’s intentions and a viewer’s perception, yielding unique, individualized meanings. This dimension focuses on how readily viewers understand or interpret a painting’s message, themes, or meanings.

#### Memory recall

Recall, a fundamental component of episodic memory, refers to the ability to retrieve and mentally represent past events without the immediate presence of the original stimulus. Unlike recognition, recall does not depend on encountering the same or a similar item but can be triggered through associative links or contextual cues, allowing memory traces to emerge spontaneously (e.g., Anderson & Bower, 1972; Jacoby, 1991; Tulving, 1976; Tulving & Watkins, 1973). Some theorists have claimed that recall involves two stages (generate–recognize theory; e.g., Anderson & Bower, 1972), relying on both associative search and a “matching-to-sample” decision-making process. Still, like recognition, recall might involve familiarity-based processes (i.e., a sense of knowing) and recollection of qualitative details (i.e., a vivid retrieval of contextual traits), respectively (Cowell et al., 2010). Art expertise further enhances memory, allowing experts to recall more details about artworks (Vogt & Magnussen, 2007).

In the present study, this dimension does not assess memory performance for paintings presented during the task. Rather, it refers to participants’ ability to report pre-existing knowledge about the painting, such as its title, artist, style, or other relevant information. In this way, responses may reflect prior knowledge and exposure, media presence, or art expertise.

## Methods

### Participants

To ensure reliability, we targeted a minimum of 55 evaluations per painting, exceeding sample sizes adopted in prior normative studies using visual stimuli (e.g., Brodeur et al., 2010, *N* = [33, 39]; Brodeur et al., 2014, *N* = [32, 42]; Johnston et al., 2010, *N* = [25, 31]; Garrido et al., 2016, *N* = 30; Thieleking et al., 2020, *N* = [56, 130]; see also the VAPS dataset of Fekete et al., 2022, which normed its 999 paintings using 20 raters per image [with 55 images rated by all 120 participants]). This sample size enabled more stable estimates of central tendency and variability across all dimensions. Given that each participant viewed up to 24 of the 144 paintings, a total sample of 330 participants was required to meet this threshold.

Of the 376 initial respondents, four were excluded due to systematic response patterns, and 11 failed the attention check (see details in the preliminary analysis section); thus, they were not included in the following analysis.

The final sample consisted of 361 European Portuguese native speakers (52.91% male, 46.81% female; *M*_age_ = 39.35 years, *SD*_age_ = 10, age range = 18–72). A minimum of 4 years of formal education^1^ was required to ensure the necessary cognitive and linguistic skills to complete the tasks.

### Stimuli

**Fig. 1:**
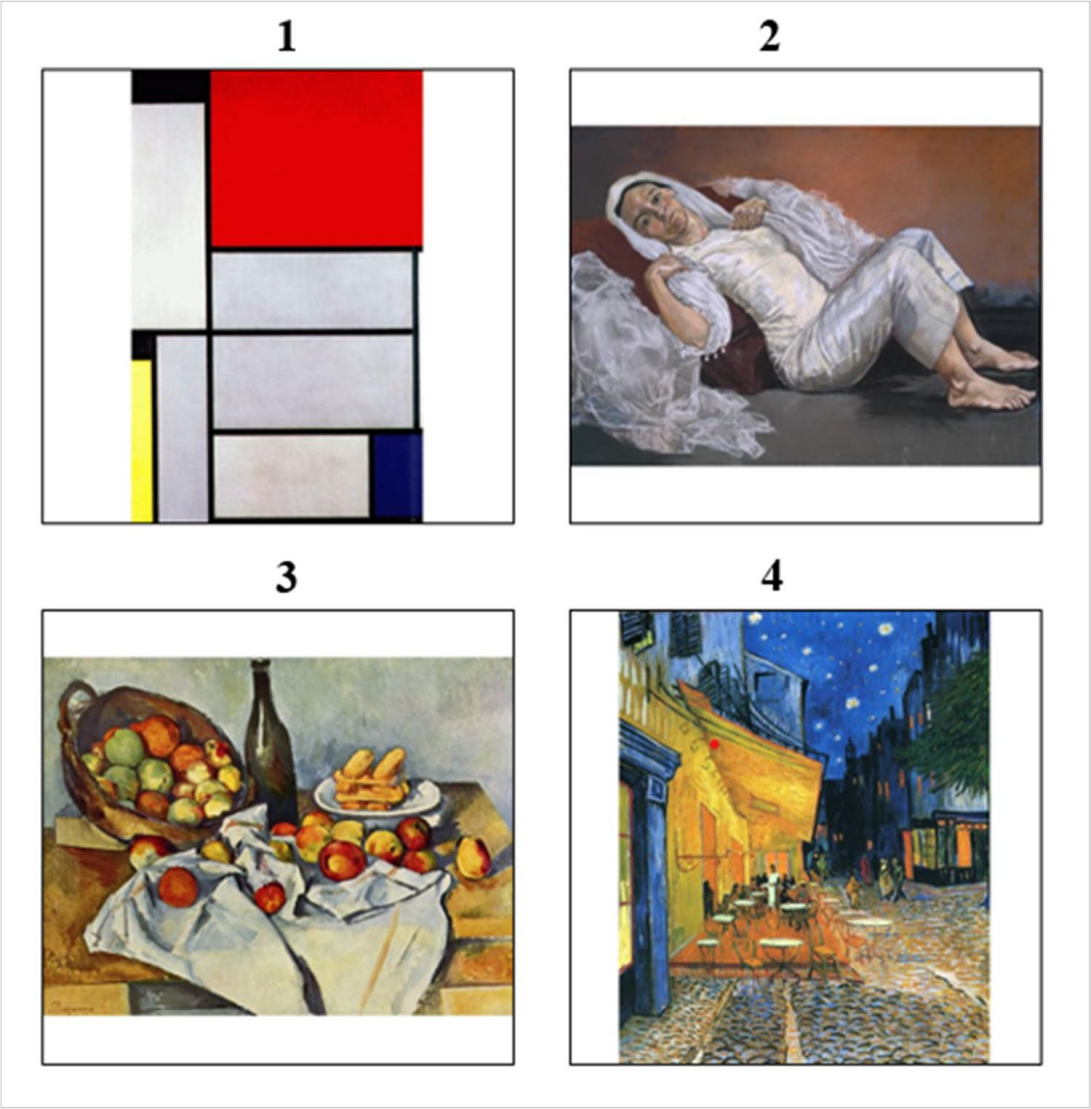
Four artworks displayed in a 2 × 2 grid with borders. *Note.* This figure displays four distinct artworks, each illustrating one of the four categories (1: *abstract*, 2: *people*, 3: *objects*, 4: *places*). Each artwork is framed with a white border to preserve its original aspect ratio, ensuring both the integrity and visual quality of the pieces

We selected 144 images of paintings from the publicly accessible WikiArt Visual Art Encyclopedia online platform. Each image was carefully standardized to a resolution of 750 × 750 pixels using IrfanView software (see Fig. 1 for examples). To maintain the original proportions (and avoid distortions), we added a white background whenever necessary. Images were adjusted for luminance (−25%) while retaining the original RGB color distribution to preserve the ecological integrity of the paintings.

Three judges selected paintings from four categories—places (*n* = 36), objects (*n* = 36), people (*n* = 36), and abstract (*n* = 36)—including well-known and lesser-known artworks to constitute a diverse range of stimuli that capture various levels of familiarity and recognition. This procedure was designed to provide a comprehensive set of stimuli applicable across a wide range of research and intervention domains.

### Procedure

This research was approved by the Research Ethics Committee of ISCTE - University Institute of Lisbon [15/2024]. Participants were recruited through online platforms (Clickworker and Prolific) and compensated at a prorated rate of €3 to €3.76 per half hour, depending on the recruitment platform. The self-paced questionnaire was implemented on the Qualtrics platform.

The study was described as an evaluation of paintings, with an expected duration of approximately 30 min. Participants were required to provide informed consent before proceeding with the study. After providing demographic information, participants were instructed to evaluate each painting in 11 dimensions using 7-point scales and respond to two open-ended questions to further assess the content-related aspects triggered by the art experience (Table 1).

**Table 1:** Dimensions and supplemental queries, their questions, and rating scales

Dimensions	Question	Scale
**Visual complexity**	Indicate how you evaluate the level of elaboration of the painting in terms of its visual details (amount of detail, interplay of lines, and patterns/number of colors).	1 (Very simple) 7 (Very complex)
**Concreteness**	Assess whether the painting represents something abstract and unrecognizable or something concrete and recognizable.	1 (Very abstract) 7 (Very concrete)
**Aesthetic appeal**	Rate how visually appealing the painting is, considering its visual characteristics and not its concept or content.	1 (Visually unpleasant/not appealing) 7 (Visually pleasant/very appealing)
**Valence**	To what extent, in your opinion, does the painting relate to something negative/unpleasant or positive/pleasant?	1 (Very negative/unpleasant) 7 (Very positive/pleasant)
**Arousal**	Rate to what extent the painting expresses something passive/calm or active/intense.	1 (Very calm) 7 (Very intense)
**Felt involvement**	Indicate your level of personal engagement, interest, and connection with the painting.	1 (Low involvement) 7 (High involvement)
**Familiarity**	Indicate how familiar the painting is to you.	1 (Not familiar) 7 (Very familiar)
**Creative inspiration**	Indicate to what extent you feel the painting stimulates or temporarily enhances your creative thinking or imagination.	1 (Not inspiring) 7 (Very inspiring)
**Memory evocation**	Indicate to what extent the painting evokes personal associations in your memory.	1 (Does not evoke personal memories) 7 (Evokes many personal memories)
**Subjective interpretation**	Indicate how easily you understand or interpret the artistic message, themes, or meaning conveyed by the painting.	1 (Very difficult) 7 (Very easy)
**Theme** **(supplemental question)**	Identify the theme or what the painting represents in your opinion (use up to 5 words).	Open-ended response
**Memory recall**	Indicate to what extent you can recall relevant information about the painting, such as its title, artist, style, or other pertinent details.	1 (No information) 7 (Much information)
**Relevant information** **(supplemental question)**	Identify the title of the painting, the artist, the style, or any relevant information associated with the painting.	Open-ended response

First, participants rated the image on nine dimensions using 7-point scales. The order of these dimensions was randomized to further control for order effects. Participants then rated the subjective interpretation dimension (7-point scale), followed by an open-ended question asking for a one-sentence description of the theme portrayed in the painting. Finally, participants responded to the memory recall dimension (7-point scale). Whenever they rated this question above 1 (indicating they recalled some information), additional fields appeared for them to provide details such as the title, artist, style, or other relevant information. If they rated the question as 1 (did not recall any information), these fields were skipped, and the participant proceeded to the following item.

The image set comprised 144 images, arranged into 24 blocks. Each block included six items of one of the four categories: people, objects, places, and abstract (six blocks per category). During the task, participants viewed one randomly selected image from each block, resulting in 24 trials per participant. The blocks were also randomized to mitigate order effects. Each image remained visible throughout its entire evaluation.

After evaluating the images, participants completed the Ten-Item Personality Inventory (TIPI, Portuguese version; Nunes et al., 2018), a brief measure of personality traits with strong convergent validity with the Big Five model (Gosling et al., 2003). We used this scale to explore the links between personality traits and the processing of artistic stimuli (e.g., Gocłowska et al., 2019; King et al., 1996). Participants rated how each trait applied to themselves using a 7-point scale (1 = strongly disagree; 7 = strongly agree). The reliability of the scale (extraversion: α = 0.707; agreeableness: α = 0.238; conscientiousness: α = 0.535; emotional stability: α = 0.595; openness to experience: α =0.526) was consistent with the original (Gosling et al., 2003) and the Portuguese validation scores (Nunes et al., 2018).

Finally, we included a five-item art experience scale to capture participants' engagement and personal involvement with the arts. The items assessed participants' appreciation for visiting art galleries and museums, the frequency of such visits, formal training in the arts (e.g., academic or nonacademic), self-identification as an artist, and their general interest and appreciation for artistic painting. Ratings were provided on a 7-point scale ranging from 1 (“totally disagree”) to 7 (“totally agree”). The scale presented good internal consistency (α = 0.783).

The questionnaire included an attention-check item embedded in the TIPI (choose option X). Upon completion of all tasks, participants were thanked and debriefed.

Following preliminary screening to ensure data quality, we assessed the consistency of the ratings across dimensions using a split-half reliability approach to determine whether the normative scores provided stable evaluations for each painting. The analytic strategy had four main goals: First, we established item-level norms for each painting across all dimensions, creating a descriptive database to support future stimulus selection and experimental control. Second, we examined whether ratings varied systematically across the four painting categories to determine whether content type shaped perceptual, emotional, and cognitive evaluations. Third, we explored the latent structure of the normative dimensions to assess whether highly correlated ratings reflected broader underlying components rather than entirely distinct constructs. Fourth, we examined associations between item-level ratings and participants’ individual characteristics to determine whether systematic variation in the evaluations could be partly explained by viewer-related factors, thereby clarifying the extent to which the ratings of the database reflect shared normative tendencies versus individual differences. In addition, the two open-ended questions were summarized as supplementary descriptive information, including the themes participants associated with each painting and the amount of accurately reported artwork-related information (e.g., title, artist, style, and other relevant details).

## Results

### Preliminary analysis

Eleven participants (2.93%) failed the attention check and were excluded from the sample. The database was also inspected for participant-level response bias. Four participants (1.12% of the sample) consistently chose the same response option at an 80% threshold. These participants were excluded. Overall, these indicators suggest a high level of engagement and reliability of the responses collected.

Outliers were identified at the item level based on ±2.5 standard deviations from the mean evaluation of each stimulus in each dimension. This analysis yielded a small number of outliers across items (0.19%) and dimensions (range 0.05–0.52%). Therefore, no responses were excluded for this reason.

We also evaluated the internal consistency of participants’ ratings across dimensions. A split-half reliability test (e.g., Prada et al., 2016), contrasting two randomly selected halves of responses within each dimension/question using *t*-tests revealed a high degree of consistency in participants' responses across the various dimensions (all *p*s > 0.865), confirming that all participants contributed equally to the normative database. The only exception was the subjective interpretation dimension, which showed a significant difference between groups (*p* =.041).

After data treatment, analyses were conducted at the item level (image) rather than the participant level. Descriptive and inferential statistics were obtained for each image in each dimension, including measures of central tendency, variability, and distribution, as well as confidence intervals.

Below, we present the descriptive statistics for the items, the results of the categorical and the thematic analysis, and the recalled details for each painting. Subsequently, we present the correlations between dimensions (per item) and their association with individual characteristics (age, gender, education, TIPI, and art experience scores).

### Item norms

The complete stimulus set (in.jpg format) and the corresponding normative database in Excel format, organized by stimulus code, are provided as supplemental material at the Open Science Framework (OSF) (https://osf.io/3mk2b/?view_only=368bffbc4f964a248ad0d1b264b2c4a9). This dataset includes detailed information for each item, such as the original database, item name, artist, and style, sourced from the original WikiArt database. For all 11 rating scales, the dataset provides averages, standard deviations, frequencies (total ratings per item), and 95% confidence intervals (CIs). Table 2 presents a summary of the descriptive statistics for each dimension.

**Table 2:** Descriptive statistics for all items in each dimension

	VC	C	AA	V	A	FI	F	CI	ME	SI	MR
*M*	4.39	4.38	4.46	4.39	4.06	3.89	3.30	3.99	2.98	3.84	2.11
*SEM*	0.06	0.12	0.06	0.06	0.07	0.05	0.09	0.06	0.07	0.11	0.06
*SD*	0.72	1.46	0.76	0.74	0.83	0.65	1.06	0.72	0.78	1.29	0.73
*R*	3.47	4.76	3.50	3.29	3.42	2.96	5.32	3.06	3.37	4.31	4.18
*MIN*	2.83	1.41	2.57	2.63	2.60	2.41	1.37	2.28	1.49	1.31	1.16
*MAX*	6.30	6.17	6.07	5.92	6.02	5.37	6.69	5.34	4.85	5.62	5.34
*Sk*	0.18	−0.69	−0.25	−0.13	0.13	−0.12	0.80	−0.26	0.35	−0.55	1.84
*SE Sk*	0.20	0.20	0.20	0.20	0.20	0.20	0.20	0.20	0.20	0.20	0.20
*K*	−0.36	−0.97	−0.33	−0.48	−0.85	−0.55	0.54	−0.53	−0.34	−1.09	3.79
*SE K*	0.40	0.40	0.40	0.40	0.40	0.40	0.40	0.40	0.40	0.40	0.40
*95% CI Upper*	4.51	4.62	4.59	4.51	4.20	4.00	3.47	4.11	3.10	4.05	2.23
*95% CI Lower*	4.28	4.14	4.34	4.27	3.93	3.78	3.12	3.87	2.85	3.62	1.99
*t-test (midpoint)*	6.58	3.11	7.37	6.32	0.90	−2.06	−7.96	−0.17	−15.83	−1.52	−31.28
*p value*	<.001^***^	.002^**^	<.001^***^	<.001^***^	.368	.041^*^	<.001^***^	.867	<.001^***^	.130	<.001^***^

*VC* = visual complexity; *C* = concreteness; *AA* = aesthetic appeal; *V* = valence; *A* = arousal; *FI* = felt involvement; *F* = familiarity; *CI* = creative inspiration; *ME* = memory evocation; *SI* = subjective interpretation; *MR* = memory recall. Abbreviations correspond to the dimensions analyzed in the study. The table presents *M* = mean; *SE* = standard error; *SD* = standard deviation; *MIN* = minimum; *MAX* = maximum; *Sk* = skewness; *SE Sk* = standard error of skewness; *K* = kurtosis; *SE K* = standard error of kurtosis; *CI* = confidence interval (95% confidence intervals for the lower and upper bounds); *t-test (midpoint)* = one-sample *t*-test against the scale midpoint

*p* <.05^*^,* p* <.01^**^, p <.001^***^

Overall, the means varied across most dimensions and were significantly different from the scale midpoint (*p* <.05). The dimensions of visual complexity, concreteness, aesthetic appeal, and valence were consistently rated above the midpoint. In contrast, ratings for felt involvement, familiarity, memory evocation, and memory recall were significantly below the midpoint of the scale. Arousal, creative inspiration, and subjective interpretation did not differ from the scale midpoint.

**Fig. 2:**
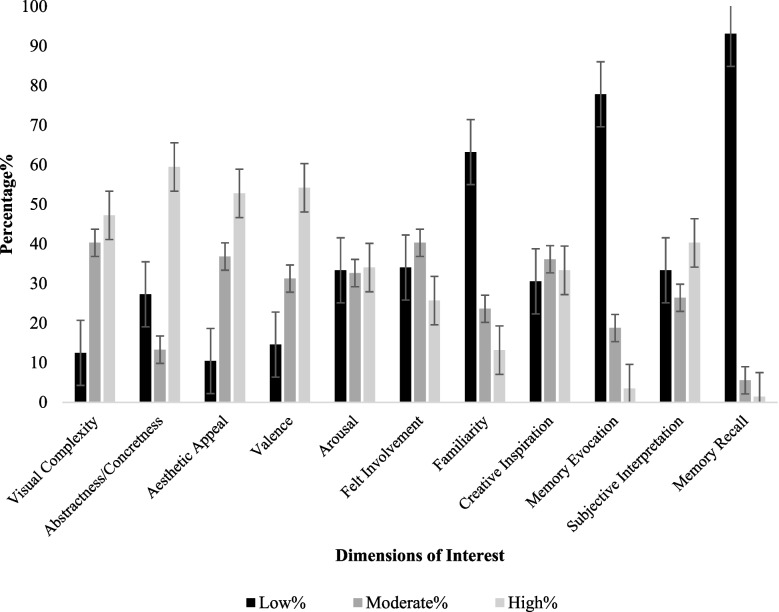
Frequency distribution of ratings across dimensions (%). *Note.* Figure 2 summarizes how paintings were distributed across low, moderate, and high levels on each dimension based on their confidence intervals. The figure shows that some dimensions, such as visual complexity, concreteness, aesthetic appeal, and valence, were skewed toward higher ratings, whereas familiarity, memory evocation, and memory recall were concentrated in the low range. Other dimensions, including arousal, felt involvement, creative inspiration, and subjective interpretation, showed a more balanced distribution of ratings across the scale. These distributions indicate which dimensions offer broad discriminatory potential for future stimulus selection and which reflect more constrained response ranges

The CIs were used to categorize stimuli as low, moderate, or high on each dimension (Prada et al., 2016; Rodrigues et al., 2018; Souza et al., 2021). Specifically, items were classified as “moderate” when the CI included the scale's midpoint (4), as “low” when the upper bound was below 4, and as “high” when the lower bound was above 4 (Fig. 2).

The normative evaluation of paintings across the 11 dimensions revealed three distinct patterns. The first comprises visual complexity, concreteness, aesthetic appeal, and valence. In this group, most paintings were rated as high (47.22–59.44%), followed by moderate (13.29–40.28%), and low (10.4–27.27%). The second includes arousal, felt involvement, creative inspiration, and subjective interpretation. These dimensions showed a more balanced distribution across high (25.69–40.28%), moderate (26.39–40.28%), and low (30.56–34.03%) ratings. Finally, familiarity, memory evocation, and memory recall received predominantly low ratings (63.19–93.06%), followed by moderate (5.56–23.61%) and high ratings (1.39–13.19%). Overall, these results suggest that while paintings are generally perceived as complex, concrete, aesthetically appealing, and positive, they are unfamiliar and trigger few mnemonic associations.

### Analysis per category

A one-way multivariate analysis of variance (MANOVA) was conducted to examine whether image category (people, objects, places, abstract) influenced participants’ evaluations across the 11 dimensions. The multivariate test revealed a significant overall effect of category, Wilks’ Λ =.073, *F*(33, 383.71) = 16.67, *p* <.001. Follow-up univariate ANOVAs confirmed significant category effects on all 11 dimensions (all *p*s <.001), and Bonferroni-corrected post hoc tests are presented in Table 3.

**Table 3:** Descriptive statistics for all items in each dimension per category

		*M*	*SEM*	*SD*	*R*	Min	Max	Sk	*SE* Sk	K	*SE* K	95% CI Upper	95% CI Lower	*F*	*p*
VC	PEO	4.33^a^	0.08	0.49	1.87	3.46	5.34	0.279	0.39	−0.51	0.77	4.50	4.17	9.01	<.001
	OBJ	4.08^a^	0.11	0.66	2.90	2.85	5.75	0.353	0.39	−0.05	0.77	4.31	3.87		
	PLA	4.30^a^	0.10	0.62	2.58	3.05	5.63	−0.02	0.39	−0.52	0.77	4.50	4.09		
	ABS	4.86^b^	0.14	0.83	3.47	2.83	6.30	−0.61	0.39	−0.10	0.77	5.14	4.58		
C	PEO	5.37^a^	0.11	0.64	2.60	3.57	6.17	−1.46	0.39	1.59	0.77	5.59	5.15	203.51	<.001
	OBJ	4.67^b^	0.13	0.81	3.25	2.73	5.98	−0.28	0.39	−0.71	0.77	4.94	4.39		
	PLA	5.32^a^	0.10	0.59	2.20	3.91	6.12	−0.73	0.39	−0.27	0.77	5.52	5.12		
	ABS	2.16^c^	0.08	0.46	1.73	1.41	3.14	0.51	0.39	−0.36	0.77	2.31	2.00		
AA	PEO	4.58^b^	0.10	0.58	2.28	3.41	5.69	0.31	0.39	−0.81	0.77	4.77	4.38	32.29	<.001
	OBJ	4.37^b^	0.10	0.60	2.51	2.92	5.43	−0.39	0.39	−0.07	0.77	4.57	4.16		
	PLA	5.13^a^	0.09	0.53	2.33	3.74	6.07	−0.64	0.39	0.42	0.77	5.31	4.95		
	ABS	3.78^c^	0.10	0.62	2.36	2.57	4.93	−0.32	0.39	−0.77	0.77	3.99	3.57		
V	PEO	4.39^a^	0.12	0.70	2.74	2.86	5.61	0.07	0.39	−0.86	0.77	4.63	4.16	22.18	<.001
	OBJ	4.37^a^	0.10	0.61	2.61	2.85	5.47	−0.52	0.39	0.08	0.77	4.58	4.17		
	PLA	4.99^b^	0.10	0.59	2.72	3.20	5.92	−0.65	0.39	0.88	0.77	5.19	4.79		
	ABS	3.80^c^	0.09	0.56	1.99	2.63	4.61	−0.46	0.39	−0.55	0.77	3.99	3.62		
A	PEO	4.30^b^	0.11	0.68	2.75	3.20	5.95	0.53	0.39	0.25	0.77	4.53	4.07	15.51	<.001
	OBJ	3.74^c^	0.12	0.75	2.74	2.72	5.47	0.54	0.39	−0.62	0.77	3.10	3.49		
	PLA	3.59^c^	0.13	0.79	2.54	2.60	5.14	0.54	0.39	−1.10	0.77	3.86	3.33		
	ABS	4.61^a^	0.11	0.68	2.83	3.18	6.02	−0.24	0.39	−0.63	0.77	4.85	4.38		
FI	PEO	4.22^a^	0.08	0.45	1.76	3.25	5.02	−0.1	0.39	−0.90	0.77	4.38	4.07	32.62	<.001
	OBJ	3.73^b^	0.09	0.54	2.48	2.89	5.37	0.97	0.39	1.00	0.77	3.92	3.55		
	PLA	4.32^a^	0.08	0.47	2.02	3.22	5.24	−0.19	0.39	−0.30	0.77	4.47	4.16		
	ABS	3.28^c^	0.09	0.55	2.27	2.41	4.68	0.41	0.39	0.05	0.77	3.46	3.09		
F	PEO	3.83^a^	0.20	1.23	4.34	2.36	6.69	0.96	0.39	−0.23	0.77	4.24	3.41	14.88	<.001
	OBJ	3.34^b^	0.16	0.96	3.40	2.17	5.57	0.76	0.39	−0.33	0.77	3.67	3.02		
	PLA	3.56^ab^	0.12	0.65	2.52	2.44	4.97	0.17	0.39	−0.37	0.77	3.78	3.35		
	ABS	2.44^c^	0.13	0.80	3.87	1.37	5.25	1.51	0.39	2.95	0.77	2.72	2.17		
CI	PEO	4.29^a^	0.07	0.52	1.87	3.45	5.32	0.15	0.39	−0.77	0.77	4.47	4.12	36.4	<.001
	OBJ	3.84^b^	0.09	0.55	2.52	2.64	5.16	−0.01	0.39	0.01	0.77	4.03	3.66		
	PLA	4.53^a^	0.08	0.51	2.05	3.29	5.34	−0.58	0.39	0.03	0.77	4.71	4.36		
	ABS	3.29^c^	0.1	0.60	2.31	2.29	4.60	0.21	0.39	−0.84	0.77	3.49	3.08		
ME	PEO	3.28^a^	0.11	0.65	2.51	2.34	4.85	0.84	0.39	0.00	0.77	3.50	3.06	27.58	<.001
	OBJ	3.03^a^	0.11	0.69	2.56	1.88	4.43	0.17	0.39	−0.40	0.77	3.27	2.80		
	PLA	3.39^a^	0.11	0.65	2.42	2.39	4.81	0.64	0.39	−0.25	0.77	3.62	3.17		
	ABS	2.19^b^	0.08	0.48	2.46	1.48	3.95	1.43	0.39	3.95	0.77	2.35	2.03		
SI	PEO	4.54^a^	0.11	0.65	2.75	2.87	5.62	−0.36	0.39	−0.04	0.77	4.76	4.32	132.75	<.001
	OBJ	4.07^b^	0.14	0.87	3.31	2.07	5.38	−0.52	0.39	−0.61	0.77	4.36	3.77		
	PLA	4.76^a^	0.10	0.61	2.21	3.34	5.55	−0.95	0.39	−0.16	0.77	4.97	4.56		
	ABS	1.97^c^	0.08	0.47	2.22	1.31	3.53	1.40	0.39	2.34	0.77	2.13	1.81		
MR	PEO	2.48^a^	0.16	0.98	4.00	1.34	5.34	1.32	0.39	1.06	0.77	2.81	2.15	6.84	<.001
	OBJ	2.20^ab^	0.11	0.68	2.61	1.35	3.96	0.94	0.39	0.05	0.77	2.43	1.96		
	PLA	1.95^bc^	0.06	0.36	1.70	1.41	3.10	1.58	0.39	2.99	0.77	2.07	1.83		
	ABS	1.80^c^	0.09	0.57	3.10	1.16	4.26	2.64	0.39	9.26	0.77	1.10	1.61		

Dimensions are coded as follows: *VC* = visual complexity; *C* = concreteness; *AA* = aesthetic appeal; *V* = valence; *A* = arousal; *FI* = felt involvement; *F* = familiarity; *CI* = creative inspiration; *ME* = memory evocation; *SI* = subjective interpretation; *MR* = memory recall. Categories: PEO = people, OBJ = objects, PLA = places, ABS = abstract paintings. The table presents *M* = mean; *SE* = standard error; *SD* = standard deviation; *MIN* = minimum; *MAX* = maximum; *Sk* = skewness; *SE Sk* = standard error of skewness; *K* = kurtosis; *SE K* = standard error of kurtosis; *CI* = confidence interval (95% confidence intervals for the lower and upper bounds)

Means that do not share the same superscript differ significantly at *p* <.05

Overall, images of people and places tended to receive higher ratings across most dimensions, namely concreteness, felt involvement, creative inspiration, and subjective interpretation, but also visual complexity, aesthetic appeal, valence, familiarity, and memory evocation. Alongside people and places, objects were rated high in visual complexity, memory evocation, and memory recall. Abstract images consistently received the lowest scores across most dimensions, except for visual complexity and arousal (Ishai et al., 2007; Murphy et al., 2012). Overall, these category-dependent differences align with prior evidence that animate and scene-related stimuli benefit from richer emotional and mnemonic engagement (e.g., Brady et al., 2008; Nairne et al., 2013).

### Qualitative descriptions

#### Artwork themes

The thematic content each participant assigned to the artworks was analyzed qualitatively. Thematic patterns varied systematically across image categories. Object-based stimuli elicited predominantly symbolic, functional, and emotionally grounded interpretations, often related to domesticity, routine, or material culture (e.g., “broken cello, furniture, earthquake” - *Bed, Chair and Bedside Table Ferociously Attacking a Cello* by Salvador Dalí); “life in the countryside” – *The Cows* by Van Gogh). Person-based images were frequently associated with relational and emotional themes, including intimacy, identity, and sociocultural roles (e.g., “either an angry couple or a sick wife” - *Hotel Bedroom* by Lucian Freud; “a humble family meal of the working class” - *The Potato Eaters* by Van Gogh). Place-related images tended to evoke memories, travel, and a sense of belonging, alongside broader narratives of rural and urban life or historical context (e.g., “river, church, it reminds me of Germany” - *A View of the Montelbaanstoren Amsterdam* by Cornelis Vreedenburgh; “travelers, North Africa” - *Arab City* by Wassily Kandinsky). Abstract stimuli elicited diverse metaphorical, emotional, and philosophical interpretations, reflecting greater variability in meaning attribution (e.g., “it conveys a sense of something fluid and in motion, somewhat intense and acidic” - *Abstract Picture* by Gerhard Richter; “a labyrinth with no way out” - *Sad Young Man in a Train* by Marcel Duchamp).

Details regarding the themes analysis and complete results for each image can be found in the supplemental material.

#### Accuracy of the analysis of recalled information

Among the 8,414 responses (rated from 1 to 7), 3,289 (39.09%) were rated above 1, indicating some recall of information about the painting. The obtained 4,366 (51.89%) written inputs were independently evaluated for the accuracy of the attributed titles, artists, styles, and other relevant details. In the pre-analysis of accuracy, responses were categorized as either correct or incorrect. For example, the correct title for a painting by Salvador Dalí would be *The Persistence of Memory*, whereas an incorrect title might be *The Passing of Time*. The same procedure was applied to artists (e.g., incorrectly attributing *The Birth of Venus* by Sandro Botticelli to Leonardo da Vinci) and to style classifications (e.g., stating that *Head of a Peasant Woman* by Vincent van Gogh represents the Impressionist movement rather than the Realist movement). For other types of information, correctness was likewise determined. For instance, identifying *Guernica* by Pablo Picasso as being housed in the *Museo Reina Sofía* was coded as correct, whereas localizing it in the Louvre museum was coded as incorrect. In the end, 2,553 (58.47%) were coded as accurate.

To examine the effects of response type and category on the number of accurate responses, we conducted a mixed ANOVA.^2^ This analysis revealed significant main effects of response type, *F*(3, 417) = 41.94, *p* <.001, η^2^ =.109, and category, *F*(3, 139) = 3.90, *p* =.010, η^2^ =.036. Notably, the interaction between response type and category was significant, *F*(9, 417) = 8.14, *p* <.001, η^2^ =.063.

Planned comparisons indicated a higher number of accurate responses for artist information (*M* = 7.49, *SE* = 0.80) than style (*M* = 5.72, *SE* = 0.43), title (*M* = 3.21, *SE* = 0.49), and other information (*M* = 1.47, *SE* = 0.21); (all *p*s <.007).

The main effect of category indicated that accurate responses were highest for people (*M* = 6.24, *SE* = 0.79), followed by objects (*M* = 5.26, *SE* = 0.78), abstract (*M* = 3.49, *SE* = 0.78), and places (*M* = 2.90, *SE* = 0.78). Significant differences in accuracy were observed between people and places (*p = *.003), between people and abstract (*p = *.014), and between objects and places (*p* =.034).

The interaction effect indicates that for title information, accuracy was significantly higher for people (*M* = 6.51, *SE* = 1.00) than for objects (*M* = 3.47, *SE* = 0.99), places (*M* = 1.69, *SE* = 0.99), and abstract stimuli (*M* = 1.14, *SE* = 0.99) (all *p*s* < *.032). For artist information, accuracy was higher for both people (*M* = 11.06, *SE* = 1.62) and objects (*M* = 10.86, *SE* = 1.60) than for abstract (*M* = 4.22, *SE* = 1.60) and places (*M* = 3.83, *SE* = 1.60). The difference was not significant between people and objects (*p = *.932) and abstract and places (*p = *.864). In contrast, for style information, accuracy was significantly higher for abstract stimuli (*M* = 8.08, *SE* = 0.85) than for objects (*M* = 5.94, *SE = *0.85), people (*M* = 5.03, *SE* = 0.86), and places (*M* = 3.81, *SE* = 0.85). These differences were significant only between abstract and people (*p = *.013) and between abstract and places (*p* <.001). Finally, the accuracy of other information was significantly higher for people (*M* = 2.34, *SE* = 0.43) than for objects (*M* = 0.75, *SE* = 0.43) and abstract stimuli (*M* = 0.53, *SE* = 0.43). Places (*M* = 2.25, *SE = *0.43) were also significantly higher than objects and abstract stimuli, and objects were significantly higher than abstract stimuli (all *p*s <.014). In contrast, the differences between people and places (*p = *.879) and between objects and abstract stimuli (*p = *.714) were not significant. Descriptive statistics for the interaction are reported in Table 4.

**Table 4:** Estimated marginal means for the category × type of information interaction

Type of information	Category	*M*	*SE*	95% CI Lower	95% CI Upper
Title	People	6.51	1.00	4.54	8.49
	Objects	3.47	0.99	1.52	5.42
	Places	1.69	0.99	−0.25	3.64
	Abstract	1.14	0.99	−0.81	3.09
Artist	People	11.06	1.62	7.85	14.26
	Objects	10.86	1.60	7.70	14.02
	Places	3.83	1.60	0.67	7.00
	Abstract	4.22	1.60	1.06	7.39
Style	People	5.03	0.86	3.32	6.73
	Objects	5.94	0.85	4.26	7.63
	Places	3.81	0.85	2.12	5.49
	Abstract	8.08	0.85	6.40	9.76
Other information	People	2.34	0.43	1.49	3.20
	Objects	0.75	0.43	−0.09	1.59
	Places	2.25	0.43	1.40	3.09
	Abstract	0.53	0.43	−0.32	1.37

Values are estimated marginal means (*M*), standard errors (*SE*), and 95% confidence intervals. Categories: people (1), objects (2), places (3), abstract (4)

Overall, artist-related information yielded the highest accuracy, followed by style, title, and other information. Category effects were comparatively modest at the main-effect level but became pronounced when examined in interaction with response type. Specifically, people-related paintings showed a clear advantage for title, artist, and other information, whereas abstract paintings were associated with higher accuracy for style recall. In contrast, accuracy for other types of information remained generally low and highly category-dependent. These findings indicate that memory recall accuracy for painting-related information varies systematically as a function of the type of information requested and the artwork's semantic category.

### Association between dimensions

The analysis of the Pearson correlations^3^ between the 11 rated dimensions reveals several very strong associations (*r* ≥ 0.80) among key dimensions.

Concreteness was correlated with subjective interpretation, suggesting that more concrete images tend to support richer personal interpretations. Aesthetic appeal correlated with valence, felt involvement, and creative inspiration, suggesting that participants tended to rate visually appealing paintings as more positive, engaging, and creatively stimulating. Likewise, valence was associated with aesthetic appeal and creative inspiration, indicating that overall positive experience appraisal of the paintings stimulates creative thinking.

Felt involvement also correlated with aesthetic appeal, creative inspiration, and memory evocation, suggesting that engaging paintings are those that are aesthetically appealing, inspiring, and evocative of personal memories. Creative inspiration also showed correlations with aesthetic appeal and valence, as well as with felt involvement and memory evocation, reinforcing the idea that a positive experience with the art and a sense of personal involvement with an artwork are closely linked to the emergence of creative thoughts.

Familiarity was associated with memory evocation and memory recall, indicating that when viewers feel they know the painting, they are more likely to evoke personal associations and recall relevant information. Memory evocation was associated with felt involvement, familiarity, and creative inspiration, suggesting that engagement, familiarity, and inspiration trigger more personal associations in memory. Pearson correlations among the dimensions of interest are presented in Table 5.

**Table 5:** Pearson’s correlation analysis of the dimensions of interest

	1	2	3	4	5	6	7	8	9	10	11
**1. VC**	–										
**2. C**	−.403^***^	–								C	
**3. AA**	−.114	.606^***^	–	AA		AA		AA			
**4. V**	−.226^**^	.529^***^	.938^***^	–				V			
**5. A**	.740^***^	−.454^***^	−.460^***^	−.597^***^	–						
**6. FI**	.175^*^	.602^***^	.802^***^	.680^***^	−.060	–		FI	FI		
**7. F**	−.051	.530^***^	.715^***^	.659^***^	−.187^*^	.758^***^	–		F		F
**8. CI**	.078	.609^***^	.906^***^	.810^***^	−.220^**^	.932^***^	.749^***^	–	CI		
**9. ME**	−.146	.682^***^	.778^***^	.740^***^	−.339^***^	.817^***^	.884^***^	.801^***^	–		
**10. SI**	−.394^***^	.939^***^	.679^***^	.630^***^	−.462^***^	.681^***^	.648^***^	.682^***^	.797^***^	–	
**11. R**	.097	.245^**^	.439^***^	.393^***^	.045	.564^***^	.864^***^	.515^***^	.661^***^	.365^***^	–

*VC* = visual complexity; *C* = concreteness; *AA* = aesthetic appeal; *V* = valence; *A* = arousal; *FI* = felt involvement; *F* = familiarity; *CI* = creative inspiration; *ME* = memory evocation; *SI* = subjective interpretation; *R* = memory recall. Abbreviations correspond to the dimensions analyzed in the study

* *p* <.05, ** *p* <.01, *** *p* <.001

### Influence of personality traits, experience with the arts, gender, age, and education on the rated dimensions

Emerging evidence suggests that stable individual differences, such as personality traits and prior art experience, influence viewers’ emotional, cognitive, and interpretive responses to visual artworks, including emotional engagement, memory, and creative insight (Afhami & Mohammadi-Zarghan, 2018; Fayn et al., 2015; Ishiguro & Okada, 2020; Sherman et al., 2015; Van Paasschen et al., 2015).

The correlation results between personal traits and the rated dimensions revealed weak but significant positive correlations. Openness to experience was positively correlated with all the dimensions except for visual complexity, concreteness, and arousal. Likewise, extraversion was positively associated with affective-related dimensions (valence, arousal, felt involvement) and memory-related dimensions (familiarity, memory evocation, and memory recall). Conscientiousness was significantly and positively correlated with subjective interpretation. Emotional stability was negatively correlated with arousal. These results suggest that personality traits are modestly but systematically related to how individuals evaluate artworks.

Art experience was also associated with most evaluative dimensions. Notably, we observed moderate correlations with felt involvement, familiarity, creative inspiration, and subjective interpretation. These associations indicate that individuals with greater art experience tend to exhibit more positive evaluative appraisals of artworks across most dimensions.

Gender showed weak positive associations with visual complexity and arousal, indicating that female participants tended to rate the paintings as slightly more visually complex and emotionally arousing. Age showed weak negative associations with felt involvement, familiarity, and memory evocation, suggesting that older participants felt somewhat less engaged and less familiar with the artworks, while displaying a weak positive correlation with subjective interpretation. In contrast, education level did not show any meaningful associations with the assessed dimensions. Pearson correlations between the dimensions of interest and individual characteristics are presented in Table 6.

**Table 6:** Pearson’s correlation analysis of the dimensions of interest with individual characteristics

Variable	VC	C	AA	V	A	FI	F	CI	ME	SI	R
**Art experience**	.087	.095	.236^***^	.188^***^	.153^**^	.317^***^	.310^***^	.338^***^	.286^***^	.308^***^	.242^***^
**Extraversion**	.052	.017	.039	.111^*^	.151^**^	.123^*^	.162^**^	.088	.194^***^	.094	.115^*^
**Agreeableness**	.029	−.031	−.005	.064	−.023	.054	−.064	.032	−.072	–.001	−.008
**Conscientiousness**	.010	.070	−.005	.072	−.078	.063	.015	.057	.019	.139^**^	.044
**Emotional stability**	−.055	.028	.009	.049	−.110*	−.011	−.072	−.051	−.030	.066	−.024
**Openness to experience**	.046	.099	.203^***^	.188^***^	.070	.273^***^	.231^***^	.257^***^	.206^***^	.209^***^	.150^**^
**Gender**	.112^*^	−.023	−.034	.022	.179^***^	−.007	.049	.057	.014	−.056	.035
**Age**	.001	.099	−.036	−.003	−.048	−.135^*^	−.147^**^	−.070	−.137^**^	.114^*^	−.062
**Education**	.064	−.024	−.054	−.057	.066	−.039	.022	−.050	−.015	.052	−.056

VC = visual complexity; C = concreteness; AA = aesthetic appeal; V = valence; A = arousal; FI = felt involvement; F = familiarity; CI = creative inspiration; ME = memory evocation; SI = subjective interpretation; R = memory recall. Abbreviations correspond to the dimensions analyzed in the study

* *p* <.05, ** *p* <.01, *** *p* <.001

## Discussion

The present database introduces a standardized set of painting stimuli that were presented and rated consistently across perceptual, emotional, and cognitive dimensions. Taken together, the normative ratings and inferential analyses offer a coherent picture of how the paintings are evaluated across dimensions and categories.

As expected, participants generally perceived paintings as visually complex, concrete, aesthetically appealing, and positive. Dimensions related to arousal, felt involvement, creative inspiration, and subjective interpretation occupied an intermediate range, echoing findings that aesthetically powerful artworks can evoke inspiration and imaginative engagement (Welke et al., 2023). Familiarity, memory evocation, and memory recall remained consistently low. These patterns converge with findings from standardized image sets (e.g., Lisbon Symbol Database [LSD]; Prada et al., 2016), which report high ratings on concreteness, aesthetic appeal, and valence, along with moderate arousal. However, our results indicate that artworks are rated as less familiar and more complex than symbols. In comparison with previous painting norms, our ratings converge in visual complexity, arousal (VAPS; Fekete et al., 2022), and valence (VAPS; Fekete et al., 2022 art.pics; Thieleking et al., 2020). Still, the observed familiarity ratings seem slightly higher than those reported in VAPS, whereas arousal levels exceeded those observed in art.pics.

Category analyses revealed that people- and place-based images tend to cluster along several evaluative dimensions, but not in visual complexity, arousal, and memory recall. People- and place-related paintings appeared to elicit greater memorability (familiarity, memory evocation, and memory recall), likely due to their richer episodic and autobiographical content, drawing upon contextually grounded representations that integrate perceptual and affective cues from personal experience (Caramazza & Shelton, 1998; Farah et al., 1989). Abstract images received the lowest ratings in most dimensions, except for the perceptually dependent dimension of visual complexity and arousal. These patterns are consistent with their lack of concrete referents and reduced potential for associative recall (Murphy et al., 2012). The absence of explicit semantic content may limit the capacity of abstract images to evoke personal associations, emotional resonance, or interpretive engagement, which may account for their reduced ratings on interpretive and memory-related dimensions (Ishai et al., 2007). Ratings of paintings depicting objects showed more variability across dimensions.

The observed recall patterns reveal that memory for artworks is selectively modulated by the interaction between semantic category and the type of information retrieved. Artist information was recalled most accurately across categories, consistent with the idea that authorship functions as a salient and stable semantic anchor in art memory. Category effects emerged primarily at the interaction level: paintings depicting people facilitated retrieval of titles, artist attributions, and other contextual details, in line with evidence that socially relevant stimuli support richer semantic encoding (Farah et al., 1989; Warrington & Shallice, 1984). In contrast, abstract paintings were associated with higher accuracy for stylistic judgments, suggesting a shift toward perceptual and formal processing when representational or narrative content is reduced, with greater reliance on features such as color, texture, and composition (e.g., Ishai et al., 2007; Murphy et al., 2012). Place-related paintings did not show a generalized accuracy advantage. Instead, they exhibited selective effects depending on the type of information accessed, indicating that scene-based representations may support certain forms of contextual processing without consistently enhancing memory accuracy. Together, these results underscore that accuracy in recalling information about art depends on both what is depicted and the nature of the knowledge being retrieved.

### Interplay between perception, emotion, and cognition

Overall, the evaluated dimensions were correlated. Within the perceptual domain, our results are consistent with previous norms of symbols (e.g., LSD; Prada et al., 2016), with concreteness positively correlated with familiarity and negatively correlated with visual complexity. Valence was positively associated with both familiarity and aesthetic appeal. As expected, visual complexity correlated positively with arousal (Souza et al., 2021) and negatively with concreteness (Prada et al., 2016). However, contrary to previous reports, visual complexity was neither negatively associated with familiarity (Brodeur et al., 2012; Prada et al., 2016; Souza et al., 2021) nor positively associated with aesthetic appeal (Souza et al., 2021). Aesthetic appeal was positively correlated with familiarity, but not with visual complexity (McDougall & Reppa, 2008). Visual complexity was again correlated with arousal, but not with familiarity (VAPS; Fekete et al., 2022). Finally, arousal was also positively correlated with valence (VAPS; Fekete et al., 2022), and valence, in turn, was correlated with both aesthetic appeal and familiarity (Prada et al., 2016).

The present findings reinforce models proposing that art engagement arises from the interplay of perceptual, cognitive, and emotional processes (Chatterjee, 2003; Leder et al., 2004; Vartanian & Skov, 2014). The strong associations between aesthetic appeal, valence, felt involvement, and creative inspiration support the view that aesthetic experience involves not only preference but also emotional resonance and interpretative engagement (Chatterjee & Vartanian, 2014). Moreover, the strong relationship between valence and creative inspiration highlights the connection between positive affect and imaginative engagement, consistent with findings that aesthetically pleasing stimuli can facilitate divergent thinking (Baas et al., 2008; Forgeard & Elstein, 2014). Likewise, the robust association between felt involvement and creative inspiration suggests that deeper personal engagement with paintings fosters ideation, supporting evidence that emotional and cognitive immersion in visual stimuli enhances creative thought (Benedek et al., 2019; Silvia, 2013). However, while previous research has linked ambiguity and cognitive challenge in artworks to deeper engagement (Muth et al., 2015; Nadal et al., 2018), our results suggest that participants were more inspired by paintings they found aesthetically appealing and emotionally pleasant, pointing to an alternative pathway to creative engagement rooted in positive affect.

The strong association between concreteness and subjective interpretation reaffirms the finding that concrete images and representations are consistently easier to interpret than abstract ones, because they anchor meaning in familiar sensory or experiential contexts (e.g., Knöchelmann et al., 2019; Pozdnyakova & Shpilnaya, 2024).

Taken together, the pattern of associations supports the idea that emotional engagement, perceived familiarity, and creative cognition are tightly linked in the context of visual art perception. These findings are consistent with previous work demonstrating that emotional salience (felt involvement) enhances memory (Kensinger, 2009; Mather & Sutherland, 2011) and creativity (Benedek et al., 2013).

Memory evocation was strongly associated with both familiarity and creative inspiration, suggesting that artworks that trigger past experiences also stimulate new ideas. This aligns with the notion that creativity often involves retrieving and recombining prior knowledge in novel ways (e.g., Benedek et al., 2013).

### Individual characteristics correlations

Prior studies have emphasized openness to experience as a key predictor of art engagement (Chamorro-Premuzic et al., 2010; Furnham & Walker, 2001). Extraversion was associated with valence, arousal, felt involvement, familiarity, memory evocation, and memory recall; conscientiousness was associated with subjective interpretation; and emotional stability was negatively associated with arousal. Openness to experience was associated with several dimensions, including aesthetic appeal, valence, felt involvement, familiarity, creative inspiration, memory evocation, subjective interpretation, and memory recall. These findings suggest that personality systematically influences how artworks are evaluated, but these associations are modest.

Moderate associations were found between art experience and felt involvement, familiarity, creative inspiration, and subjective interpretation, while weaker associations were found with aesthetic appeal, valence, arousal, memory evocation, and memory recall. This pattern indicates that individuals with greater experience and exposure to art tend to evaluate paintings as more engaging, familiar, and creatively inspiring, and to interpret them more richly. These findings support the view that cultivated interest and prior exposure shape aesthetic responses more robustly than personality traits alone (Chamberlain et al., 2019; Van Paasschen et al., 2015).

Overall, gender and age showed only weak associations with the ratings, while education displayed no meaningful relationship with any dimension.

## Implications and future directions

Overall, our findings align with principles established for standardized stimuli, including objects (e.g., Souza et al., 2021), symbols (e.g., Snodgrass & Vanderwart, 1980), and emotion-eliciting images (e.g., Libkuman et al., 2007). However, paintings, unlike simpler stimuli, evoke complex patterns of affect, memory, and meaning-making, shaped by cultural and historical context (Chatterjee & Vartanian, 2014; Pelowski et al., 2017).

The current findings also highlight the significant role of semantic image categories in shaping subjective evaluations and cognitive-affective responses to visual art. These differences align with previous research suggesting that figurative and scenic images convey greater emotional and semantic richness, thereby facilitating narrative engagement and personal relevance for viewers (Cupchik et al., 2009; Vessel et al., 2012). This difference across painting categories may reflect the lower semantic anchoring of abstract content, which, despite its visual novelty, offers fewer associative cues for memory and meaning construction (Leder et al., 2004).

Future studies could expand on this work by systematically manipulating the associative richness or narrative potential of images within and across categories to determine their causal impact on creative ideation and aesthetic experience. The Vienna Integrated Model of Aesthetic Appreciation and Aesthetic Judgments highlights the interplay between perceptual, cognitive, and emotional processes in shaping aesthetic responses, suggesting that image categories may differentially activate these mechanisms (Leder et al., 2004).

Although this dataset constitutes an important resource for research on aesthetic perception, a few limitations warrant consideration. Future studies should directly examine the predictive validity of the present norms by testing whether specific dimensions predict independent behavioral, attentional, or neural outcomes (e.g., paintings rated as more familiar or less visually complex may be expected to produce faster responses). The image set was not curated using art-historical criteria, such as stylistic lineage, compositional structure, or thematic conventions, which limits the interpretability of cross-painting comparisons. Although we had anticipated that the open-ended responses might provide richer insight into mnemonic and semantic associations, most were brief and descriptive; accordingly, these materials were retained as supplementary rather than emphasized as a central contribution. In addition, our sample consisted exclusively of Portuguese participants from a WEIRD (Western, educated, industrialized, rich, and democratic) population (Henrich et al., 2010), and cross-cultural variation in aesthetic judgment may therefore be underestimated. The use of an online testing environment also reduces control over viewing conditions and may introduce variability in attention and engagement. Addressing these limitations will require curated image corpora grounded in art history, cross-cultural validation samples, and controlled laboratory testing. Equally crucial, combining behavioral ratings with neurophysiological methods (e.g., electroencephalography [EEG], eye-tracking, or neuroimaging) will enable a more mechanistic account of how visual art shapes perceptive, affective, and cognitive processing.

## Conclusion

By standardizing a set of paintings and providing normative data, this study bridges a methodological gap in aesthetics research. The database offers a unified framework for investigating aesthetic perception, creativity, and memory. Findings reinforce established cognitive-affective principles while capturing the complexity of fine art as a stimulus.

The database also provides a useful methodological resource by allowing researchers to select matched subsets of paintings while limiting variation in potentially confounding attributes. This may support more controlled and reproducible painting-based research. More broadly, the norms may prove useful across a range of behavioral, attentional, neural, cross-cultural, and intervention-oriented contexts.

## Data Availability

All data and materials are available on the Open Science Framework (OSF): https://osf.io/3mk2b/?view_only=368bffbc4f964a248ad0d1b264b2c4a9

## Footnotes

1 This criterion is frequently adopted in normative and cognitive aging studies to ensure sufficient literacy and comprehension skills for task performance (e.g., Souza et al., 2021).

2 Mauchly’s test indicated that the assumption of sphericity was violated for the within-subjects factor; therefore, Greenhouse–Geisser-corrected statistics are reported.

3 The strength of the observed Pearson correlations was interpreted according to Cohen's criteria (Cohen, 1988), as follows: correlations from 0.10 to 0.29 represent “weak associations,” from 0.30 to 0.49 indicate “moderate associations,” from 0.50 to 0.79 indicate “strong associations,” and values of 0.80 and above represent “very strong associations.”
